# An Incidental Finding of Anemia: Rectal Adenocarcinoma in a Young Adult

**DOI:** 10.7759/cureus.4554

**Published:** 2019-04-27

**Authors:** Waqas Memon, Michael Reno, Chriss Mulumba

**Affiliations:** 1 Internal Medicine, Carle Foundation Hospital, Urbana, USA; 2 Internal Medicine, University of Illinois at Urbana-Champaign, Champaign, USA; 3 Psychiatry, Carle Foundation Hospital, Urbana, USA

**Keywords:** adenocarcinoma, internal medicine, oncology, young adult

## Abstract

Rectal adenocarcinoma is an uncommon finding in patients under the age of 40. However, epidemiological surveys have suggested that colorectal cancers are increasing in incidence among patients aged 20 to 39 years in the United States. Colorectal adenocarcinoma is often not considered in the differential diagnosis in this demographic because of age. Here, we present the case of an incidental finding of anemia during a preliminary evaluation of rheumatoid arthritis leading to the diagnosis of stage IV-B rectal adenocarcinoma in a 34-year-old male patient.

A 34-year-old Caucasian male presented with the incidental finding of anemia during a preliminary evaluation for rheumatoid arthritis. The patient was asymptomatic with the exception of a three-month history of wrist and ankle joint pain. Past medical history was positive for only a three-year history of occasional spotty, painless rectal bleeding attributed to internal hemorrhoids. Physical exam findings were positive for mild extremity pallor and positive fecal occult blood test. Hematologic studies revealed a significant microcytic, hypochromic anemia with severe iron deficiency. Laboratory studies revealed no evidence of vitamin deficiency, hemolytic activity, hematuria, hypothyroidism, or clotting factor disorder. Erythrocyte sedimentation rate (ESR), rheumatoid factor, and cyclic citrullinated peptide 3 (CCP3) were elevated supporting the diagnosis of underlying rheumatoid arthritis. On further questioning, the patient revealed that he had been utilizing an average of 2000 mg of ibuprofen daily during the previous several months in an attempt to control his joint pain. The patient was evaluated for a potential upper gastrointestinal bleed by esophagogastroduodenoscopy (EGD), which found no evidence of active bleeding. As the patient continued to have decreasing hemoglobin levels, he was evaluated for a lower gastrointestinal source of bleeding by colonoscopy, which revealed an 8 cm circumferential mass at the anal verge. Pathological evaluation of biopsy samples revealed a moderately differentiated invasive adenocarcinoma. The patient had no family history of colorectal cancer or major associated risk factors, such as obesity, smoking history, heavy alcohol use, diabetes mellitus type 2, or a history of inflammatory bowel disease. Following discharge, positron emission tomography (PET) scan showed extensive metastatic disease to multiple regional lymph nodes as well as multiple suspicious hepatic lesions and bilateral pulmonary nodules. Due to the poor prognosis, recommended treatment consisted of folinic acid, 5-fluorouracil, oxaliplatin (FOLFOX-4) along with palliative radiation.

The cause of the increase in the incidence rate of colorectal cancer in young adults remains unknown. Among this demographic, colorectal cancers appear to be more aggressive and present at later stages with more advanced disease. In young adults, the most common clinical sign at presentation is rectal bleeding. In young adults presenting with seemingly common gastrointestinal complaints, a high degree of suspicion for colorectal cancer may be warranted by clinicians.

## Introduction

Rectal adenocarcinoma is an uncommon finding in patients under the age of 40 [1,2]. However, epidemiological surveys have suggested that colorectal cancers are increasing in incidence among patients aged 20 to 39 years in the United States [3,4]. Colorectal adenocarcinoma is often not considered in the differential diagnosis in this demographic because of age. Here, we present the case of an incidental finding of anemia during a preliminary evaluation of rheumatoid arthritis leading to the diagnosis of stage IV-B rectal adenocarcinoma in a 34-year-old male patient.

## Case presentation

**Figure 1:**
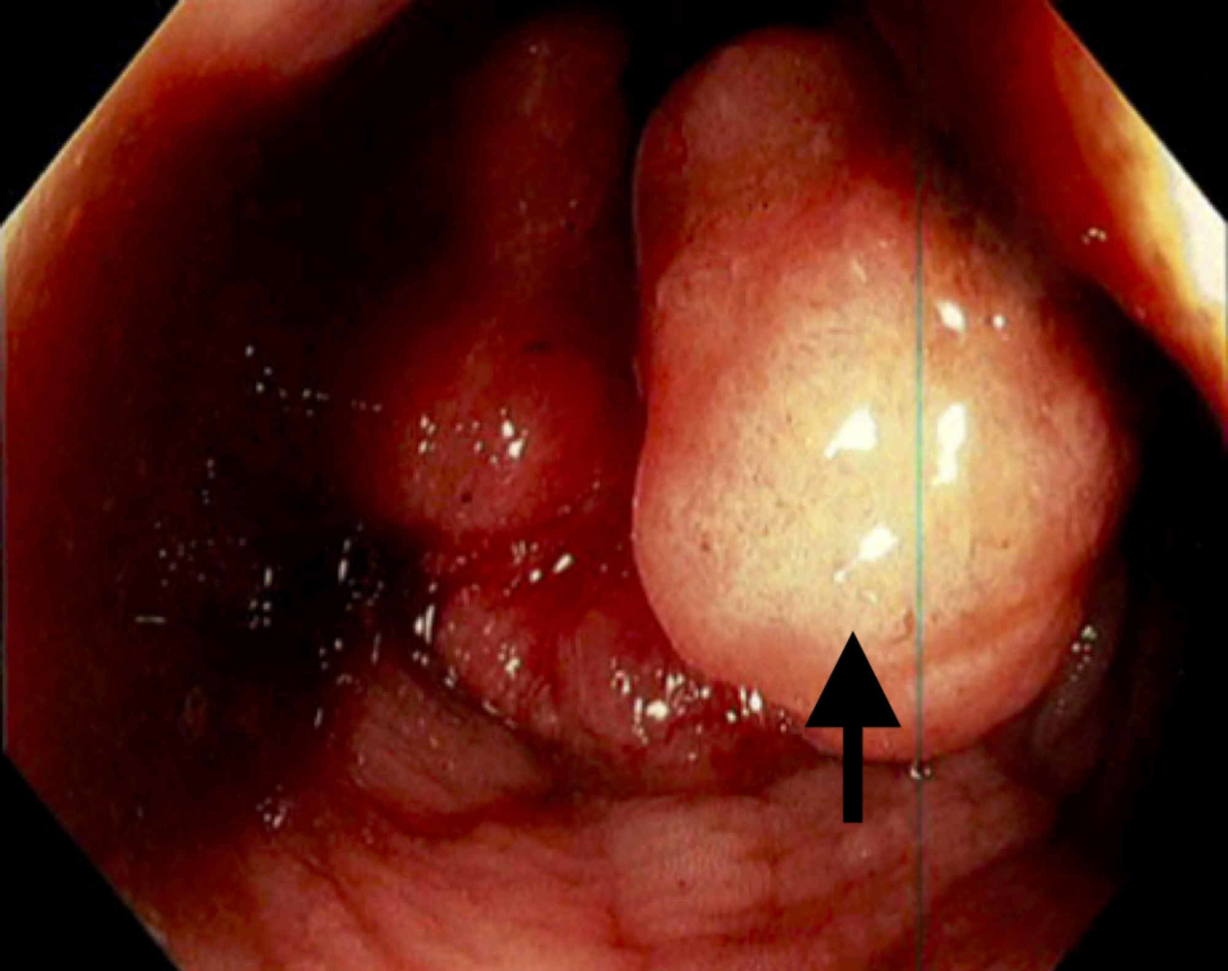
Fungating rectal mass 8 cm proximal to the anal verge extending to the anal verge, circumferential in diameter.

A 34-year-old Caucasian male presented with the incidental finding of anemia during a preliminary evaluation for rheumatoid arthritis. The patient was asymptomatic with the exception of a three-month history of wrist and ankle joint pain. Past medical history was positive for only a three-year history of occasional spotty, painless rectal bleeding attributed to internal hemorrhoids. Physical exam findings were positive for mild extremity pallor and positive fecal occult blood test. Hematologic studies revealed a significant microcytic, hypochromic anemia with severe iron deficiency. Laboratory studies revealed no evidence of vitamin deficiency, hemolytic activity, hematuria, hypothyroidism, or clotting factor disorder. Erythrocyte sedimentation rate (ESR), rheumatoid factor, and cyclic citrullinated peptide 3 (CCP3) were elevated supporting the diagnosis of underlying rheumatoid arthritis. On further questioning, the patient revealed that he had been utilizing an average of 2000 mg of ibuprofen daily during the previous several months in an attempt to control his joint pain. He was evaluated for a potential upper gastrointestinal bleed with an esophagogastroduodenoscopy (EGD), which found no evidence of active bleeding. As the patient continued to have decreasing hemoglobin levels, he was evaluated for a lower gastrointestinal source of bleeding by colonoscopy, which revealed an 8 cm circumferential mass at the anal verge (Figure 1).

Pathological evaluation of biopsy samples revealed a moderately differentiated invasive adenocarcinoma. The patient had no family history of colorectal cancer or major associated risk factors, such as obesity, smoking history, heavy alcohol use, diabetes mellitus type 2, or a history of inflammatory bowel disease. Following discharge, positron emission tomography (PET) scan showed extensive metastatic disease to multiple regional lymph nodes as well as multiple suspicious hepatic lesions and bilateral pulmonary nodules. Due to the poor prognosis, recommended treatment consisted of folinic acid, 5-fluorouracil, oxaliplatin (FOLFOX-4) along with palliative radiation.

## Discussion

In young adults, the most common clinical sign at presentation is rectal bleeding. A retrospective study indicated in men and women aged 20 to 49 years there has been a 1.5% per year increase and a 1.6% per year increase in colorectal carcinoma (CRC) from 1992 to 2005 [5]. In patients with newly diagnosed CRC for patients aged 20-49 years, it is reported that the incidence will double by 2030, and inversely there will be a decline in the incidence of CRC by more than one-third among patients older than the screening age of 50 years [6]. In 2010, this demographic was diagnosed with 4.8% of all colon and 9.5% of all rectal cancers; it is predicted that by the year 2030 this will increase to 10.9% and 22.9%, respectively [6]. Among this demographic, colorectal cancers appear to be more aggressive and present at later stages with more advanced disease [7,8].

This increasing incidence of CRC is alarming and highlights the need for a screening modality and modification of behavioral factors. Options for routine screening in CRC include stool-based tests: guaiac-based fecal occult blood test (gFOBT), highly sensitive fecal immunochemical test (FIT), and multi-targeted stool DNA test (MT-sDNA); structural exams: Colonoscopy, flexible sigmoidoscopy (FSIG) and computed tomography (CT) colonography (virtual colonoscopy). High-risk patients are advised to begin screening prior to the age of 50, whereas patients younger than 50 without risk factors are recommended against undergoing routine screening [8]. The observed increasing incidence of CRC is hypothesized to be attributed to behavioral factors which include obesity, physical inactivity, and the western diet [8]. Physical activity has been proven to reduce the risk for both colon and rectal cancers, with odds ratios of 0.70 and 0.51, respectively [9]. The combination of all these factors identifies modifiable risk factors in the younger patient population.

As stated previously, the incidence rate of CRC will increase by 2030. Given this data, education regarding risk factors, and improving a healthy lifestyle will be very crucial [5]. Given that there is an absence of routine screening, a joint effort between the patient and health care professional will be critical in minimizing delays in both diagnosis and treatment.

## Conclusions

In young adults, rectal adenocarcinoma is usually diagnosed later and is associated with a poor prognosis. There is unclear etiology but the early incidence could be associated with modern dietary factors and obesity. Although rectal carcinoma is a rare finding in young male patients, there is no screening guideline available. An appropriate history and physical is crucial in the diagnosis along with a family history which is important for surveillance. Particularly relevant is a family history of a young family member or a first degree relative with rectal carcinoma. Multiple treatment modalities are present but overall the patients have a poor prognosis.

In conclusion, young patients presenting with seemingly common gastrointestinal complaints, a high degree of suspicion for colorectal cancer may be warranted by clinicians.
